# *G1/ELE* Functions in the Development of Rice Lemmas in Addition to Determining Identities of Empty Glumes

**DOI:** 10.3389/fpls.2016.01006

**Published:** 2016-07-12

**Authors:** Mengjia Liu, Haifeng Li, Yali Su, Wenqiang Li, Chunhai Shi

**Affiliations:** ^1^State Key Laboratory of Crop Stress Biology for Arid Areas, College of Agronomy, Northwest A&F University, YanglingChina; ^2^Xinjiang Agricultural Vocational Technical College, ChangjiChina; ^3^College of Agriculture and Biotechnology, Zhejiang University, HangzhouChina

**Keywords:** rice, empty glume, lemma, *G1/ELE*, *OsLHS1*

## Abstract

Rice empty glumes, also named sterile lemmas or rudimentary lemmas according to different interpretations, are distinct from lemmas in morphology and cellular pattern. Consistently, the molecular mechanism to control the development of lemmas is different from that of empty glumes. Rice *LEAFY HULL STERILE1*(*OsLHS1*) and *DROOPING LEAF*(*DL*) regulate the cellular pattern and the number of vascular bundles of lemmas respectively, while *LONG STERILE LEMMA1* (*G1*)/*ELONGATED EMPTY GLUME* (*ELE*) and *PANICLE PHYTOMER2* (*PAP2*)/*OsMADS34* determine identities of empty glumes. Though some progress has been made, identities of empty glumes remain unclear, and genetic interactions between lemma genes and glume genes have been rarely elucidated. In this research, a new *G1/ELE* mutant *g1–6* was identified and the phenotype was analyzed. Similar to previously reported mutant lines of *G1/ELE*, empty glumes of *g1–6* plants transform into lemma-like organs. Furthermore, Phenotypes of single and double mutant plants suggest that, in addition to their previously described gene-specific functions, *G1/ELE* and *OsLHS1* play redundant roles in controlling vascular bundle number, cell volume, and cell layer number of empty glumes and lemmas. Meanwhile, expression patterns of *G1/ELE* in *osmads1-z* flowers and *OsLHS1* in *g1–6* flowers indicate they do not regulate each other at the level of transcription. Finally, down-regulation of the empty glume gene *OsMADS34/PAP2* and ectopic expression of the lemma gene *DL*, in the *g1–6* plants provide further evidence that empty glumes are sterile lemmas. Generally, our findings provided valuable information for better understanding functions of *G1* and *OsLHS1* in flower development and identities of empty glumes.

## Introduction

Flower development is the basis for seed development in angiosperms. Based on analyses of flower mutants in *Arabidopsis thaliana* and *Antirrhinum majus*, the ABCDE model was proposed to interpret the molecular mechanism in control of flower development (Coen and Meyerowitz, 1991; Pelaz et al., 2000; Theissen, 2001; Theissen and Saedler, 2001; Ditta et al., 2004).

Rice (*Oryza sativa L.*) is a model plant of monocots and one important cereal crop. The structural units of the rice flower are spikelets and florets. The spikelet is the primary unit of the rice inflorescence and contains a fertile floret and a pair of empty glumes (also called “sterile lemmas” or “rudimentary lemmas”), subtended by a pair of highly reduced glumes called rudimentary glumes. The floret consists of a pair of bract-like organs (lemma and palea), two lodicules (equivalent to eudicot petals), six stamens, and a carpel (Yoshida and Nagato, 2011; Lombardo and Yoshida, 2015).

Recently, functions of many homeotic genes have been elucidated in rice. There are four *APETALA1* (*AP1*)/*FRUITFULL* (*FUL*) orthologs in the rice genome: *OsMADS14*, *OsMADS15*, *OsMADS18*, and *OsMADS20* (Kater et al., 2006). Recent studies suggested that *OsMADS14*, *OsMADS15*, and *OsMADS18* together with *OsMADS34/PANICLE PHYTOMER2* (*PAP2*) regulate the transition from shoot apical meristem (SAM) to the inflorescence meristem (IM; Kobayashi et al., 2012). The *AP3* homolog *SUPERWOMAN1* (*SPW1*)/*OsMADS16* is expressed in lodicules and stamens. Mutations in *SPW1* results in homeotic transformation from stamens to carpels and lodicules into lemma/palea-like structures respectively (Nagasawa et al., 2003). *OsMADS3*, *OsMADS58*, *OsMADS13*, and *OsMADS21* belong to the *AGAMOUS* (*AG*) subfamily. Among them, *OsMADS3* and *OsMADS58* are expressed in stamens and carpels. *OsMADS3* mainly functions in regulating stamen development while *OsMADS58* mainly determines the carpel development (Yamaguchi et al., 2006; Dreni et al., 2011; Li et al., 2011a). *OsMADS13* is expressed in ovules and specifies ovule identity. When mutations occurred in *OsMADS13*, ovules were transformed into carpelloid organs (Dreni et al., 2007; Li et al., 2011b; Yamaki et al., 2011). Additionally, floral determinacy is redundantly determined by *OsMADS3*, *OsMADS58*, and *OsMADS13* (Yamaguchi et al., 2006; Dreni et al., 2011; Li et al., 2011b). No obvious function of *OsMADS21* has been found (Dreni et al., 2007).

The *SEP* subfamily of rice consists of five genes. *OsMADS7* and *OsMADS8*, two *SEP3* homologs, are expressed in the inner three whorls and function in floral development redundantly (Cui et al., 2010). In addition to *OsMADS7* and *OsMADS8*, *LEAFY HULL STERILE1* (*OsLHS1*), *OsMADS5* and *OsMADS34*/*PAP2* were divided into the *LOFSEP* subgroup of *MADS*-box genes (Kobayashi et al., 2010). *OsLHS1* plays versatile roles in floral organ development. In *OsLHS1* mutant plants, lemmas, and paleas are under-developed and they fail to interlock each other. Meanwhile, lodicules and stamens develop abnormally. Additionally, the identity of floral meristem is affected. As a result, one new floret is formed in the spikelet occasionally (Jeon et al., 2000; Agrawal et al., 2005; Chen et al., 2006; Gao et al., 2010). *OsMADS5* does not have any obvious function in flower development (Agrawal et al., 2005). Like *OsLHS1*, another member of the rice *LOFSEP* subgroup *OsMADS34*/*PAP2* has versatile functions in flower development. In addition to regulating spikelet meristem identity and ovule development, *OsMADS34*/*PAP2* regulates the development of empty glumes. In *OsMADS34*/*PAP2* mutant plants, empty glumes elongate to form leaf-like or lemma-like organs (Gao et al., 2010; Kobayashi et al., 2010; Lin et al., 2014). With evolutionary and sequence analyses of *OsMADS34*/*PAP2*, Lin et al. (2014) provided evidence to support the hypothesis that rice empty glumes originated from the lemmas of degenerate florets and named them as rudimentary lemmas.

In addition to ABCDE genes, there are other homeotic genes involved in the development of rice floral organs and/or meristems. Among them are *DROOPING LEAF* (*DL*) and *OsMADS6/MOSAIC FLORAL ORGANS1 (MFO1)*. *DL* plays a key role in specifying carpel identity and regulates the number of vascular bundles in lemmas (Yamaguchi et al., 2004; Li et al., 2011a,b). *OsMADS6/MFO1*, a member of the *AGL6* subfamily, determines the palea identity and has versatile functions in regulating flower development (Ohmori et al., 2009; Li et al., 2010; Zhang et al., 2010; Yadav et al., 2011; Duan et al., 2012).

*LONG STERILE LEMMA1 (G1)* /*ELONGATED EMPTY GLUME* (*ELE*) encodes a DUF640 domain protein and determines identities of empty glumes. When mutations occurred in *G1*/*ELE*, empty glumes transformed into lemma-like organs (Yoshida et al., 2009; Hong et al., 2010). Meanwhile, natural mutations in the genome of allotetraploid *Oryza grandiglumis* cause similar homeotic conversions in empty glumes, suggesting that empty glumes are serial lemma homologs that have been modified by the action of *G1/ELE* (Yoshida et al., 2009).

Despite the fact that the molecular mechanism controlling reproductive organ development in rice is well-understood, the control of empty glume identity remains unclear. In this study, *g1–6*, a new strong mutant allele of *G1/ELE*, was identified and double mutant *g1–6 osmads1-z* plants were analyzed. Additionally, the expression profile of *DL*, *OsLHS1*, *G1/ELE*, and *OsMADS34/PAP2* was analyzed. Our findings provided valuable information for understanding functions of these genes and interpreting identities of empty glumes.

## Materials and Methods

### Plant Materials

A single recessive rice mutant, *g1–6*, displaying abnormal empty glumes was identified from a M_2_ population of 9311 (cultivar *indica*), obtained via ethylmethane sulfonate (EMS) mutagenesis (Ye et al., 2006); *osmads1-z* was identified previously (Gao et al., 2010). The 9311 cultivar was used as a wild type strain for phenotype observation and RNA extraction. All plants were planted in the greenhouse in Northwest A&F University or paddy fields in Yangling and Hang Zhou in China under natural conditions. In the greenhouse, the conditions were 14 h of light at 28°C, 10 h of dark at 22°C during the vegetative stage. The conditions were adjusted to 10 h of light at 28°C, and 14 h of dark at 22°C to induce the transition from the vegetative stage to the reproductive stage. The humidity was maintained at 70%.

### Histological Analysis and Scanning Electron Microscopy (SEM)

Spikelets of rice plants grown in a paddy field in Yangling were fixed in 75% ethanol and 25% acetate and dehydrated in a series of graded ethanol. For histological analysis, materials were substituted by xylene and embedded in paraplast plus and cut into 8 μm-thick sections. Then, sections were stained with 0.2% toluidine blue and photographed using a Nikon E600 microscope and Nikon DXM1200 digital camera. For scanning electron microscopy (SEM), samples were dehydrated in a series of ethanol solutions, then dried at a critical point, sputter-coated with platinum, and observed under a JSM-6360LV (JEOL) scanning electron microscope, as described previously (Li et al., 2010).

### RNA Extraction

Rice plants grown in a paddy field in Yangling under field conditions during the 2014 cultivation season were used. Empty glumes, lemmas, and paleas of wild type 9311, *g1–6*, *osmads1-z*, and *g1–6 osmads1-z* plants at the Sp6 stage defined by Ikeda were collected (Ikeda et al., 2004) and preserved at -80°C. Total RNA was isolated using a TRIZOL kit (Sangon Biotech) according to the manufacturer’s protocol and treated with DNaseI (Sangon Biotech). First-strand cDNA was synthesized using a Prime Script RT reagent kit (Fermentas) according to the protocol.

### Statistical Analyses

Stained 8 μm-thick sections of randomly selected samples at the Sp6 stage were observed using a Nikon E600 microscope. Data of the number of vascular bundles and thickness of different kinds of organs from different genetic backgrounds were analyzed using Duncan’s test (Duncan, 1955). Letters A, B, or C of every sample in **Figures 5** and **7** indicates a significant difference between two analyzed samples at the level of *P* < 0.01. If the letters of two samples are the same, there is no significant difference between these two samples.

### Quantitative RT-PCR

Reverse transcription polymerase chain reaction (RT-PCR) was performed using CFX96 real-time polymerase chain reaction (PCR) detection systems (Roche Applied Science). A final volume of 15 μL reactions contained 7.5 μL SYBR^®^ Premix Ex Taq (Takara, 2×), 0.75 μL forward and reverse primers (10 pM) respectively, 0.5 μL cDNA (5.0 ng/μL) and 5.5 μL ddH_2_O. PCR cycling conditions were 95°C for 30 s followed by 40 cycles of 95°C for 5 s, 55°C for 30 s and 61 cycles of 65°C for 10 s. Three biological replicates were sampled in each experiment and all samples were run with three technical replicates. Data acquisition and analyses were performed using the Roche Light Cycler software. Samples were normalized using *ACTIN* expression (Kim et al., 2003) and relative expression levels were determined using the 2(-Δ Ct) analysis method (Pfaﬄ, 2001). Primers used in this article are listed in Supplementary Table S1.

## Results

### Identification of the *g1–6* Allele

**FIGURE 1:**
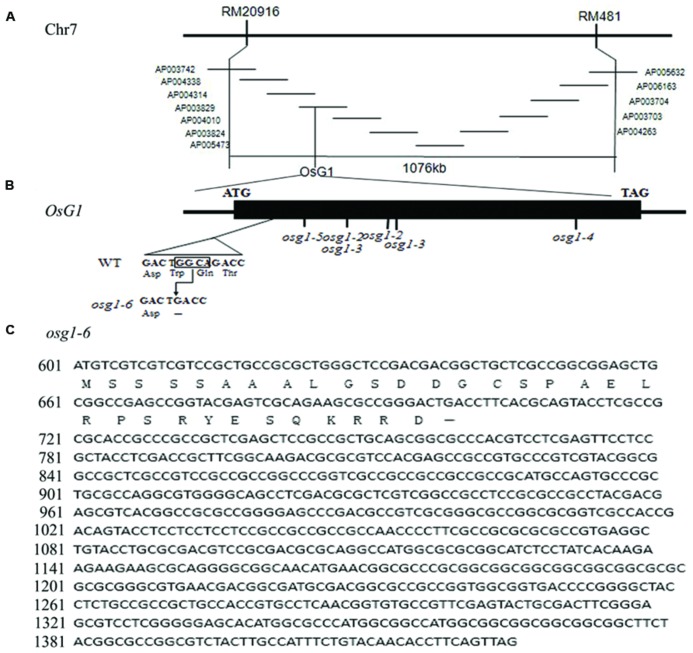
**Positional cloning of *g1–6* and sequence analysis. (A)** The candidate gene was mapped in BAC AP003829 on the short arm of Chromosome 7. **(B)** Schematic representation of *G1* and mutation site of *g1–6*. **(C)** Full cDNA of *g1–6*. The translation is terminated prematurely at the mutation site.

To identify rice mutants with floral defects, a population of rice mutants in the *indica* subspecies 9311 background treated by EMS was screened. A mutant line displaying an abnormal phenotype of empty glumes was isolated. When mutant plants were crossed with 9311 plants, the F_1_ progeny generated normal flowers, whereas the F_2_ plants yielded a ~3:1 ratio of phenotype segregation, indicating that this phenotype was linked with one nuclear recessive locus mutation. To identify the mutant gene, a map-based cloning strategy was used. The homozygous mutant plants were crossed with one *japonica* cultivar 02428. Using a population of 484 F_2_ mutant plants, the mutation site was mapped between two simple sequence repeat (SSR) molecular markers, RM20916 and RM481, on the short arm of chromosome 7 (**Figure 1A**).

Since plants of *long sterile lemma1* and *elongated empty glume* displayed similar phenotypes and the *G1/ELE* gene was mapped in the same region (Yoshida et al., 2009; Hong et al., 2010), we considered if a mutation of *G1*/*ELE* occurred in our mutant plants. Therefore, we designed a pair of primers to amplify a fragment including the encoding sequence, partial 5′ UTR and 3′ UTR of *G1/ELE*. Then PCR products using genome DNA extracted from wild-type and mutant plants as templates were sequenced respectively. Compared with the wild-type plants, a 4-bp (+98 to +101) deletion was found in *G1/ELE* of our mutant plants (**Figure 1B**). This result proved that our mutant was an allele of *G1/ELE*. The *G1/ELE* gene only contains one exon and encodes one DUF640 domain protein which is localized to the nucleus (Yoshida et al., 2009; Hong et al., 2010). In combination with its transcriptional activity, we speculated that *G1/ELE* played a role as a transcription factor. The mutation in our plants resulted in a frame shift and caused a premature termination of translation at the 33rd amino acid immediately after the deletion, and a complete loss of the DUF640 domain (**Figure 1C**). Since five mutant lines of *G1/ELE* have been reported previously (Yoshida et al., 2009; Hong et al., 2010), we named our mutant *g1–6*.

### Empty Glumes of *g1–6* Plants Transform into Lemma-Like Organs

**FIGURE 2:**
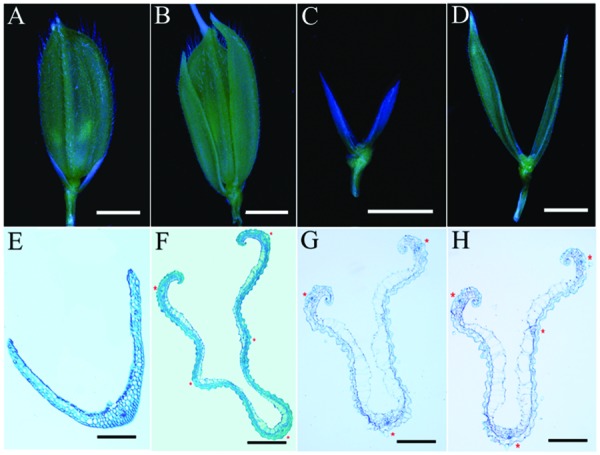
**Phenotypes of spikelets and empty glumes. (A)** One spikelet of wild type. **(B)** One *g1–6* spikelet. **(C)** One empty glume of wild type. **(D)** One *g1–6* empty glume. Transverse sections of one empty glume **(E)** and one lemma of wild type **(F)**. Transverse sections of one *g1–6* empty glume **(G)** and one *g1–6* lemma **(H)**. Red asterisks in **(F–H)** indicate vascular bundles. Bars = 2 mm in **(A–D)**, 100 μm in **(E)**, 200 μm in **(F–H)**.

**FIGURE 3:**
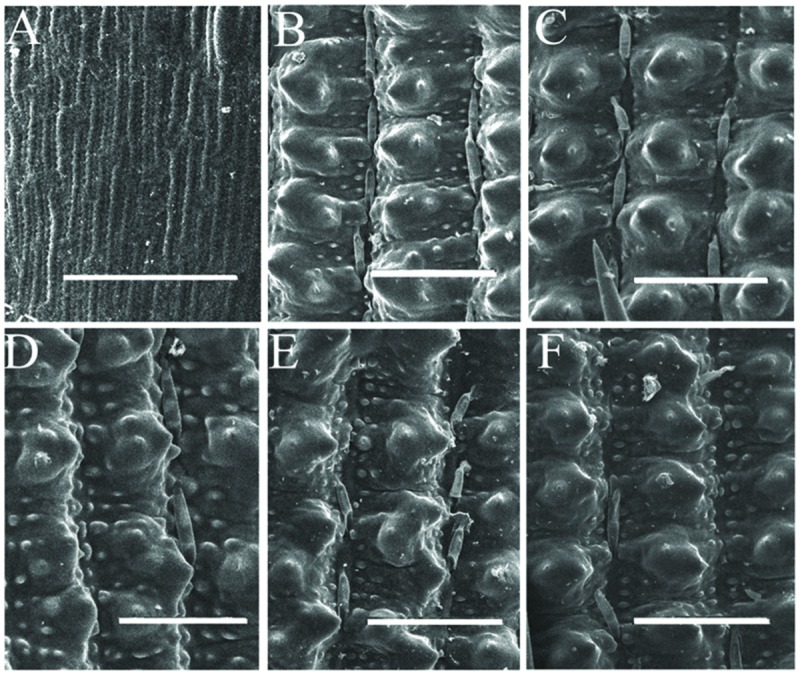
**Scanning electron microscopy observation of empty glumes, lemmas, and paleas.** Epidermis of one empty glume **(A)**, one lemma **(B)**, and one palea **(C)** of wild- type plants. Epidermis of one empty glume **(D)**, one lemma **(E)**, and one palea **(F)** of *g1–6* plants. Bars = 50 μm.

Compared with wild-type 9311 plants, mutant plants develop normally at the vegetative stage (data not shown). The flowering time of mutant plants is about 90 days after sowing (DAS), which is indistinct from the wild-type in Hangzhou. However, mutant plants displayed abnormal empty glumes during the reproductive stage (**Figures 2A,B**). Therefore, we analyzed phenotypes of empty glumes in detail. Firstly, the average length of abnormal empty glumes in *g1–6* plants was >8 mm whereas that of wild type plants is about 3 mm (**Figures 2C,D**, Supplementary Figure S1). Secondly, whereas wild-type empty glumes form only one vascular bundle (**Figure 2E**), *g1–6* empty glumes develop 3–5 vascular bundles (**Figures 2G,H**). Thirdly, similar to those of lemmas in wild type plants, two marginal regions of abnormal empty glumes in *g1–6* plants curled inwardly (**Figures 2F–H**). Fourthly, like wild-type lemmas, abnormal empty glumes contain four types of cells: silicified cells (sc), fibrous sclerenchyma (fs), spongy parenchymatous cells (spc), and no silicified cells (nsc; **Figures 2F–H**). On the contrary, wild-type empty glumes only contain one type of cell, sclerenchymatous cells, between two epidermal layers (**Figure 2E**). Furthermore, the epidermal cells of abnormal empty glumes in *g1–6* were similar to those of lemmas and paleas but differed from those of glumes of wild-type plants (**Figure 3**). In conclusion, empty glumes in *g1–6* plants transformed into lemma-like organs. These phenotypes are similar to those of the long sterile lemmas (LSLin *g1–1* plants, but stronger than those of *ele* plants (Yoshida et al., 2009; Hong et al., 2010). This is probably, because the mutation in *ele* was a one-nucleotide substitution of G to A, resulting in only one amino acid substitution of glycine to serine in the protein (Hong et al., 2010).

### *g1–6 osmads1-z* Plants Displayed Similar Defects in Inner Floral Organs and Floral Meristems to *osmads1-z*

**FIGURE 4:**
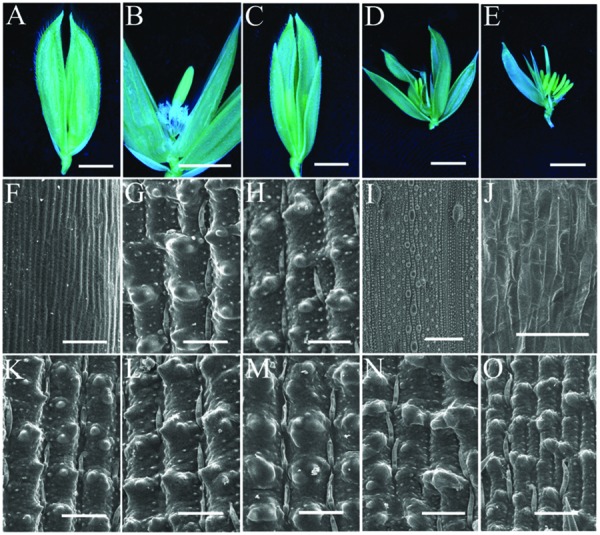
**Flower Phenotypes of *osmads1-z* and *osmads1-z g1–6* plants. (A,B)** Spikelets of *osmads1-z*. **(C–E)** Spikelets of *osmads1-z g1–6*; the lemma and palea were removed in **(E)** to show the inner organs. Epidermal cells of one empty glume **(F)**, one lemma **(G)** and one palea **(H)** of *osmads1-z*; Epidermal cells of one leaf **(I)** and one lodicule **(J)** of wild type plants. Epidermal cells of one empty glume **(K)**, one lemma **(L)** and one palea **(M)** of *g1–6 osmads1-z*. **(N)**, Epidermal cells of one lodicule of *osmads1-z*. **(O)** Epidermal cells of one lodicule of *osmads1-z g1–6*. Bars = 2 mm in **(A–E)**, 50 μm in **(F–O)** except for **(I)**, 100 μm in **(I)**.

*LHS1-*like genes are a grass specific clade of *SEP-*like genes (Malcomber and Kellogg, 2004). In grasses, homologs of *OsLHS1* display distinct expression patterns in different species, implying that differences in *LHS1* expression patterns may contribute to the morphological diversification of grass inflorescence architecture (Malcomber and Kellogg, 2004). The biological function of *OsLHS1* has been elucidated. It is mainly strongly expressed in lemmas, paleas and pistils, as well as floral meristems at the early stage (Li et al., 2010). In general, it regulates the development of lemmas/paleas and affects the meristem determinacy of inner floral organs (Jeon et al., 2000; Agrawal et al., 2005; Chen et al., 2006; Gao et al., 2010). Previously, one null *OsLHS1* mutant line *osmads1-z*, containing a 1312 bp deletion which spans the first exon and intron, was identified (Gao et al., 2010). Similar to other alleles of *OsLHS1*, lemmas and paleas in *osmads1-z* could not interlock closely (**Figure 4A**). Meanwhile, the development of lodicules and stamens was disrupted (Gao et al., 2010; **Figure 4B**), while the empty glumes developed normally (**Figures 4A,B**).

To analyze the genetic interaction between *G1/ELE* and *OsLHS1*, a double mutant was constructed and flower phenotypes of double mutant plants were analyzed. Primary observations indicated that double mutant plants exhibited additive phenotypes, i.e., the phenotype of *g1–6 osmads1-z* empty glumes was similar to that of *g1–6* plants (**Figure 4C**), whereas phenotypes of inner floral organs mimicked *osmads1-z* (**Figures 4D,E**). Like *g1–6* plants, empty glumes of *g1–6 osmads1-z* plants developed into lemma-like organs (**Figure 4C**). Correspondingly, similar to *osmads1-z*, lemmas and paleas of double mutant plants could not interlock each other (**Figures 4A,C**); meanwhile, phenotypes of the inner floral organs of *g1–6 osmads1-z* plants were the same as those of the *osmads1-z* plants mentioned above: the number of stamens decreased; development of lodicules was disrupted and the epidermal cells were different from those of wild type lodicules, but indistinct from those of *osmads1-z* plants (**Figures 4B,D,J,N,O**, Supplementary Table S2). As reported in *osmads1-z*, double mutant plants exhibited defects in floral meristems by generating ectopic florets occasionally (Gao et al., 2010; **Figure 4E**).

### The Mutation of *g1–6* Enhanced the Phenotype of Lemmas in *osmads1-z* Plants

**FIGURE 5:**
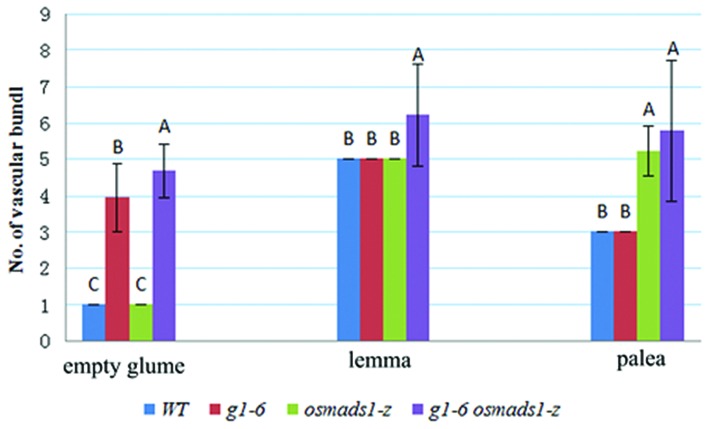
**Statistical analyses of number of vascular bundles in empty glumes, lemmas, and paleas.** For every kind of organs, 15 samples of wild-type (WT), *g1–6*, *osmads1-z*, *g1–6 osmads1-z* were examined with transverse sections, respectively. The means were statistically analyzed with the Duncan test and grouped (A, B, and C) according to significant differences at *P* < 0.01. Error bars indicate SD.

To further elucidate the function of *OsLHS1 and G1/ELE* in lemma development, we analyzed phenotypes of abnormal empty glumes and lemmas in double mutant plants in detail. Epidermal (Epidermal) cells of abnormal empty glumes were indistinct from those of *g1-6* or *osmads1-z* lemmas/paleas, but distinct from those of wild-type empty glumes (**Figures 4F–H,K,L**); similarly, epidermal cells of double mutant lemmas/paleas were indistinct from those of *g1-6* or *osmads1-z* plants, but distinct from those of wild-type leaves (**Figures 4G–I,L,M**), indicating that they still maintained partial identities of lemmas or paleas. However, two differences were found, including the number of vascular bundles, and lemma thickness. Previously, we characterized one strong *dl* mutant line and found the function of *DL* in regulating the number of vascular bundles in lemmas (Li et al., 2011b). Since the ectopic expression of *DL* was detected in this research (see below, **Figure 8**), we investigated the number of vascular bundles in empty glumes and lemmas/paleas of double mutant plants. The number of vascular bundles of abnormal empty glumes was 3.94 ± 0.93 in *g1–6* plants, whereas the number increased to 4.67 ± 0.72 in *g1–6 osmads1-z* plants. Meanwhile, the number of vascular bundles in lemmas increased from 5.00 ± 0.00 in *osmads1-z* plants to 6.2 ± 1.42 in *g1–6 osmads1-z* plants (**Figure 5**). Statistical analysis showed these differences were significant (**Figure 5**).

**FIGURE 6:**
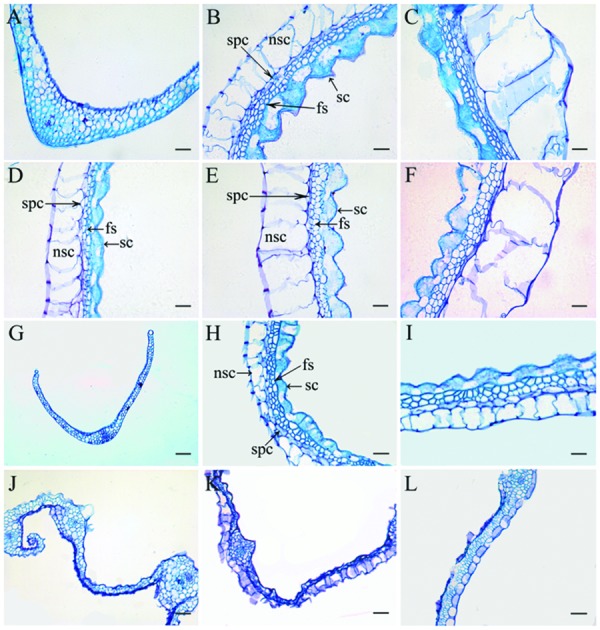
**Histological observation of empty glumes, lemmas, and paleas.** Transverse sections of one empty glume **(A)**, one lemma **(B)**, and one palea **(C)** in wild type plants. Transverse sections of one abnormal empty glume **(D)**, one lemma **(E)**, and one palea **(F)** in *g1–6* plants. Transverse sections of one empty glume **(G)**, one lemma **(H)**, and one palea **(I)** in *osmads1-z* plants; Transverse sections of one empty glume **(J)**, one lemma **(K)**, and one palea **(L)** in *osmads1-z g1–6* plants. Sc, silicified cells; fs, fibrous sclerenchyma; spc, spongy parenchymatous cells; nsc, no silicified cells. Bars = 50 μm.

**FIGURE 7:**
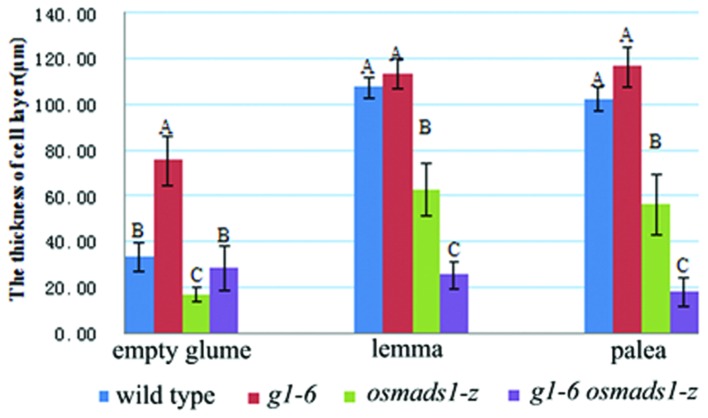
**Statistical analyses of the thickness of empty glumes, lemmas, and paleas.** For every kind of organ, 13 samples of wild type, 14 samples of *g1–6*, 17 samples of *osmads1-z*, 22 samples of *g1–6 osmads1-z* plants were measured with transverse sections, respectively. The means were statistically analyzed with the Duncan test and grouped (A, B, and C) according to significant differences at *P* < 0.01. Error bars indicate SD.

Previously, it was reported that *OsLHS1* controls the differentiation of specific cell types in lemmas/paleas (Prasad et al., 2005). Therefore, we carefully analyzed the cellular pattern of lemma-like organs and lemmas/paleas of the wild-type, *g1–6*, *osmads1-z*, and *g1–6 osmads1-z* plants at same developmental stage. Lemmas of *osmads1-z* plants were thinner than those of wild type plants. The average thickness of lemmas of *osmads1-z* plants was 62.74 ± 11.27 μm, whereas that of wild-type plants was 107.20 ± 4.81 μm (**Figures 6B,C,H,I** and **7**). We found the total size of the four cell types of lemma-like organs and lemmas/paleas in double mutant plants were further reduced in *g1–6 osmads1-z* plants than those in single mutant *osmads1-z* or *g1–6* plants (**Figures 6D–L**). Spongy parenchymatous cells were not observed in some regions (**Figures 6J–L**). As a result, the thickness of lemma-like organs and lemmas/paleas in double mutant plants was much thinner than corresponding organs in *g1–6* or *osmads1-z* plants (**Figures 6** and **7**). Statistically, these differences are meaningful (**Figure 7**).

### Expression Profile of *DL*, *OsLHS1*, *G1/ELE*, and *OsMADS34/PAP2*

To further analyze the genetic interactions between genes associated with development of empty glumes and lemmas, expression patterns of *DL*, *OsLHS1*, *G1/ELE*, and *OsMADS34* were analyzed under different genetic backgrounds by using quantitative RT-PCR.

**FIGURE 8:**
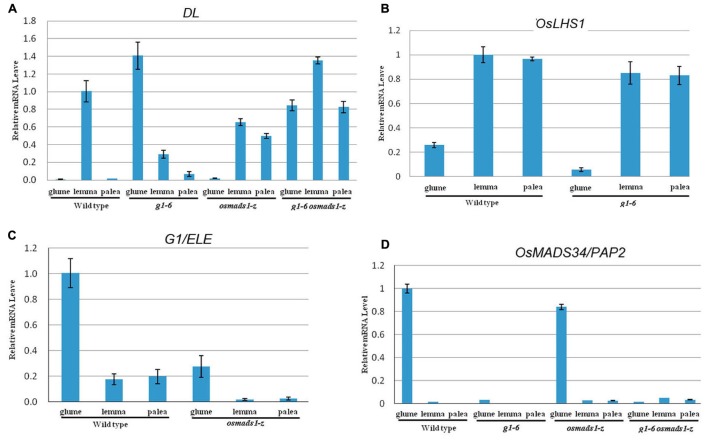
**Expression patterns of *DL*, *OsLHS1*, *OsMADS34/PAP2*, and *G1/ELE.* (A)** Expression analyses of *DL* gene in empty glumes, lemmas, and paleas. **(B)** Expression analyses of *OsLHS1* in empty glumes, lemmas, and paleas. **(C)** Expression analyses of *G1/ELE* in empty glumes, lemmas, and paleas. **(D)** Expression analyses of *OsMADS34/PAP2* in empty glumes, lemmas, and paleas. Error bars indicate SD.

In rice, *DL* is expressed in lemmas in addition to carpels, but not in paleas and empty glumes of wild-type plants (Nagasawa et al., 2003; Li et al., 2011b; **Figure 8A**). In addition to specifying carpel identity, *DL* regulates the development of vascular bundles in lemmas (Nagasawa et al., 2003; Li et al., 2011b). Based on these facts, we initially analyzed the expression pattern of *DL*. As shown in **Figure 8A**, strong ectopic expression of *DL* was detected in abnormal empty glumes of *g1–6* and double mutant plants, further suggesting empty glumes acquire identities of lemmas. Meanwhile, consistent with the increased number of vascular bundles, ectopic expression of *DL* was detected in paleas of *osmads1-z* and double mutant plants (**Figure 8A**).

In wild-type plants, *OsLHS1* was strongly expressed in lemmas and paleas, whereas the expression was weak in empty glumes (**Figure 8B**). Since empty glumes transform into lemma-like organs in *g1–6* plants, we predicted *OsLHS1* might be strongly expressed in abnormal empty glumes of *g1–6* plants. Surprisingly, the expression level of *OsLHS1* in abnormal glumes of *g1–6* plants was lower than that of lemmas or paleas (**Figure 8B**). Similarly, mutation in *osmads1-z* did not change the expression domains of *G1/ELE* (**Figure 8C**). These results indicate *OsLHS1* and *G1/ELE* do not regulate each other at the level of transcription. *OsMADS34*/*PAP2* is another regulator of empty glumes. Mutations in *OsMADS34*/*PAP2* also disrupt the development of empty glumes (Gao et al., 2010; Kobayashi et al., 2010; Lin et al., 2014). We considered if the expression of *OsMADS34*/*PAP2* was altered in *g1–6* plants and analyzed its expression. In wild-type plants, strong expression of *OsMADS34/PAP2* was detected in empty glumes but excluded from lemmas and paleas (**Figure 8D**). However, no obvious expression of *OsMADS34/PAP2* in abnormal glumes of either *g1–6* or double mutant plants was detected (**Figure 8D**). These results suggested that *G1/ELE* regulate the expression of *OsMADS34*/*PAP2* positively in empty glumes. It was reported that *OsLHS1* represses the expression of *OsMADS34*/*PAP2* (Khanday et al., 2013). However, obvious expression of *OsMADS34*/*PAP2 w*as not found in lemmas or paleas of *osmads1-z* (**Figure 8D**). One probable reason is that other genes also repress the expression of *OsMADS34*/*PAP2*.

## Discussion

### Distinct Mechanism for Controlling the Development of Empty Glumes and Lemmas

According to one hypothesis, the spikelet is a reduced leaf branch comprising a series of bracts and florets. Similar to rudimentary glumes, empty glumes have been considered as metamorphic bract leaves (Chase, 1968; Bell, 1991; Takeoka et al., 1993). The alternative hypothesis is that empty glumes are remnants of two lower reduced florets that lost all their inner floral organs. Under this interpretation, a rice spikelet would possibly have been derived from an ancestral one that contained three florets, and empty glumes would then represent the sterile lemmas rather than the empty glumes (Yoshida and Nagato, 2011). Recently, Lin et al. (2014) proposed that empty glumes were rudimentary lemmas. Relatively, the hypothesis that empty glumes are sterile lemmas is more acceptable.

At the molecular level, the mechanism to determine lemmas is also different from that of empty glumes. It was reported that *OsLHS1* controls the differentiation of specific cell types in lemmas and paleas (Prasad et al., 2005). The development of lemmas and paleas was disrupted and they could not interlock each other in *OsLHS1* mutant plants (Jeon et al., 2000; Chen et al., 2006; Gao et al., 2010). These results implied that *OsLHS1* regulates the development of lemmas. *DL* is expressed in lemmas and carpels, and regulates the number of vascular bundles of lemmas in addition to specifying identities of carpels (Yamaguchi et al., 2004; Li et al., 2011a,b). In *dl-sup6*, the number of vascular bundles in lemmas decreases (Li et al., 2011b). In *osmads6-1* or *osmads1-z* plants, *DL* is ectopically expressed in abnormal paleas which form more than three vascular bundles (Li et al., 2010, 2011a; **Figure 7**). Together, we suggest that *DL* regulates lemma identity by determining the number of vascular bundles. Its expression in lemma-like organs in *g1–6* is consistent with the transformation from empty glumes to lemmas (**Figure 8A**; Lin et al., 2014). However, mutations of neither *OsLHS1* nor *DL* caused the severe loss of lemma identities. Mutations of *OsMADS6/MFO1* and *DEP/OsMADS15* retarded the development of paleas, but lemmas developed normally (Ohmori et al., 2009; Li et al., 2010; Wang et al., 2010; Zhang et al., 2010; Duan et al., 2012). The simultaneous suppression of three *AP1/FUL-like* genes *OsMADS14*, *OsMADS15*, and *OsMADS18* did not affect the development of lemmas (Kobayashi et al., 2012), suggesting that they are not regulators of lemmas. Key regulators of lemmas might be identified in future research.

Two genes have been reported to determine the identities of empty glumes. *G1/ELE*, and *OsMADS34/PAP2*. Research (Yoshida et al., 2009; Hong et al., 2010) clearly showed *G1/ELE* was a key gene for maintaining the identities of empty glumes. Including natural mutations, empty glumes in all *g1/ele* plants lose identities. All features including the length, cell pattern, type of epidermal cells, inward curl of marginal tissues and the number of vascular bundles showed the transformation from empty glumes to lemmas (Yoshida et al., 2009; Hong et al., 2010; this research). Similarly, empty glumes of all *osmads34/pap2* plants lose identities and develop into lemma-like or indeterminate organs, suggesting *OsMADS34/PAP2* is also a key regulator for empty glume development (Gao et al., 2010; Kobayashi et al., 2010; Lin et al., 2014).

At present, there are several controversial interpretations regarding the identities of empty glumes; whether they are true glumes or lemmas. Yoshida et al. (2009) regarded empty glumes as remnants of two lower reduced florets and named them sterile lemmas. Indeed, formation of lemma-like organs at the position of empty glumes in the *Oryza grandiglumis* is probably resulted from the natural mutations in *G1/ELE* (Yoshida et al., 2009). Similarly, Lin et al. (2014) proposed empty glumes originated from lemmas and named them as rudimentary lemmas based on the analysis of *OsMADS34/PAP2* mutant plants and other research. The repression of *OsMADS34/PAP2* and ectopic expression of *DL* in lemma-like organs in *g1–6* plants (**Figure 8**) provided further molecular evidence to support these hypotheses. Probably, research progress in the development of lemmas and further molecular evidence might provide clues to determine the identities of empty glumes. The cloning and identification of key genes for lemma identities and analyses of their expression in empty glumes are necessary.

### The Relationship between *G1/ELE*, *OsMADS34/PAP2*, and *OsLHS1*

Previously, it was reported that *OsLHS1* represses the expression of *OsMADS34/PAP2*. Further analysis showed *OsMADS34/PAP2* is a direct target gene negatively regulated by *OsLHS1* (Khanday et al., 2013). *OsMADS34/PAP2* maintains the identity of the spikelet meristem, whereas *OsLHS1* specifies the identity of the floral meristem. The probable mechanism is that *OsLHS1* promotes the transition from spikelet meristem to floral meristem by repressing the expression of *OsMADS34/PAP2*. In the present research, we found that expression of *OsMADS34/PAP2* was dramatically decreased in abnormal empty glumes in *g1–6* plants (**Figure 8D**). Taking the transcriptional activity and nuclear location of the G1/ELE protein into account, we propose *OsMADS34/PAP2* is downstream of *G1/ELE.* Additionally, although the expression of *G1/ELE* is not affected on *osmads34-2*, empty glumes still lose identities (Gao et al., 2010). These results suggest that *G1/ELE* might function in the development of empty glumes by regulating the expression of *OsMADS34/PAP2*. At present, it is unclear whether *G1/ELE* regulates *OsMADS34/PAP2* directly or not; therefore, further analysis is needed for clarification.

In combination with their functions in empty glumes and lemmas respectively, phenotypes of empty glumes and inner floral organs in *g1–6 osmads1-z* plants indicate *G1/ELE* and *OsLHS1* function independently.

Compared with *osmads1-z* plants, the increased number of vascular bundles and decreased thickness of lemmas in double mutant plants (**Figures 6** and **7**) showed that the abnormality becomes more severe. The enhancement of phenotypes suggests *G1/ELE* plays a role in the development of lemmas redundant with *OsLHS1*. Evidently, the redundancy is unequal, and although the expression of *OsLHS1* is weak in abnormal glumes of *g1–6* plants, the thickness is normal. This result implies low level expression of *OsLHS1* is enough to maintain the normal cellular pattern (thickness) of lemma-like organs. On the contrary, in *osmads1-z* plants, although *G1/ELE* was still expressed in lemmas at low level, the thickness was decreased.

The participation of *G1/ELE* in lemma development further provided evidence to support the statement that empty glumes are sterile lemmas.

Sequence data from this article can be found in the GenBank/EMBL data libraries under accession numbers AB106553 (*DL*), AK100227 (*OsMADS34/PAP2*), AK070981 (*OsLHS1*), AB512480 (*G1/ELE*).

## Author Contributions

Experimental design: HL, CS; Experiments: ML, HL, YS, WL, CS; Data analysis: HL, ML; Manuscript preparation: HL, ML; Supervision, funding and reagents: HL, CS.

## Conflict of Interest Statement

The authors declare that the research was conducted in the absence of any commercial or financial relationships that could be construed as a potential conflict of interest.
