# Supplementary figures and images for: G1/ELE Functions in the Development of Rice Lemmas in Addition to Determining Identities of Empty Glumes
Source: Front Plant Sci. 2016 Jul 12;7:1006. doi: 10.3389/fpls.2016.01006 (PMC4941205; doi:10.3389/fpls.2016.01006)

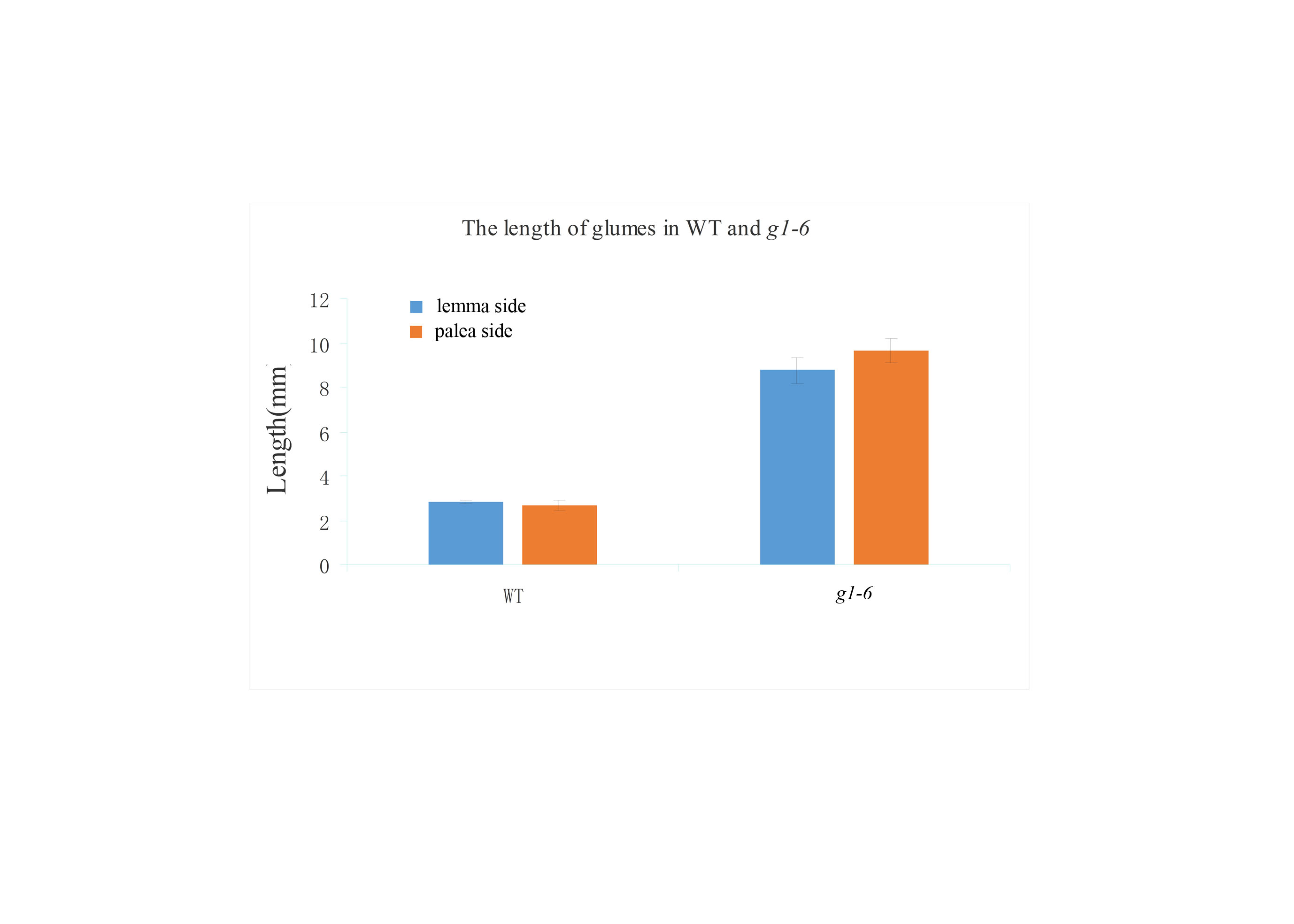

Supplement: Supplementary file 1 [file Image_1.JPEG]
