# Supplementary material for: G1/ELE Functions in the Development of Rice Lemmas in Addition to Determining Identities of Empty Glumes
Source: Front Plant Sci. 2016 Jul 12;7:1006. doi: 10.3389/fpls.2016.01006 (PMC4941205; doi:10.3389/fpls.2016.01006)
Supplement: Supplementary file 2 [file Table_1.PDF]

Supplementary Material

G1 and *LHS1* Determine the Identity of Glumes and Lemmas Synergistically in Rice

Meng-jia Liu[1], Ya-li Su[1], Wen-qiang Li[1], Chun-hai Shi[2]*, Haifeng-Li[1, 3]*

*** Correspondence:** Dr. Haifeng-Li: lhf@nwsuaf.edu.cn; Prof. Chun-hai Shi: chhshi@zju.edu.cn

Supplementary Table 1. Number of floral organs

Floral organs	**Average number of organs**			
	WT	*osg1*	*osmads1*	*osg1 osmads1*
Normal or abnormal glume	2	2	2	2
Lemma-like or palea-like organs*	0	0	4.38 ±0.80	4.05 ±0.55
Lodicule organs	2	2	0	0
Stamens	6	6	3.03 ±1.75	3.91 ±4.19
Carpel	1	1	1.43 ±0.67	1.86 ±1.33

A total of 72 *osmads1* and 77 *osmads1 osg1* spikelets were examined.

*Lemma and palea in first whorl are not included.
