# Supplementary material for: G1/ELE Functions in the Development of Rice Lemmas in Addition to Determining Identities of Empty Glumes
Source: Front Plant Sci. 2016 Jul 12;7:1006. doi: 10.3389/fpls.2016.01006 (PMC4941205; doi:10.3389/fpls.2016.01006)
Supplement: Supplementary file 3 [file Table_2.PDF]

Supplementary Material

G1 and *LHS1* Determine the Identity of Glumes and Lemmas Synergistically in Rice

Meng-jia Liu[1], Ya-li Su[1], Wen-qiang Li[1], Chun-hai Shi[2]*, Haifeng-Li[1, 3]*

*** Correspondence:** Dr. Haifeng-Li: lhf@nwsuaf.edu.cn; Prof. Chun-hai Shi: chhshi@zju.edu.cn

Supplementary Table 2. Primers used in this paper

Primer name	Sequence (5'~3')
G1-F	TTCAGGTAGCCCCTACCTAGCTA
G1-R	GAAGCAACGGAACGAACACG
OsACTINQPF	TGCTATGTACGTCGCCATCCAG
OsACTINQPR	AATGAGTAACCACGCTCCGTCA
G1QPF	CGCGGCATCTCCTATCACAA
G1QPR	CGCTCCCGAAGTCGCAGTA
OsMADS1QPF	CCTCAAACAAATCAGGTCAAGAAA
OsMADS1QPR	ATACCCAATCTGCAGGGAATG
DLQPF	CCAAGCCAGATATCCCTCACA
DLQPR	CTTCACCGCCGATATAGATCG
OsMADS34QPF	GTTCCTCTCTCCTTCCTCTTCAA
OsMADS34QPR	AAAAGGATGGTTGTCCCGCT
